# 1-[2-(3,4-Dichloro­phen­yl)-5-(3,4,5-trimethoxy­phen­yl)-2,3-dihydro-1,3,4-oxadiazol-3-yl]ethanone

**DOI:** 10.1107/S1600536808020771

**Published:** 2008-07-09

**Authors:** Dao-Hang He, Yong-Chuang Zhu

**Affiliations:** a School of Chemistry and Chemical Engineering, South China University of Technology, Guangzhou 510640, People’s Republic of China

## Abstract

The title compound, C_19_H_18_Cl_2_N_2_O_5_, was synthesized by the reaction of *N*′-(3,4-dichloro­benzyl­idene)-3,4,5-trimethoxy­benzo­hydrazide and acetic anhydride. The oxadiazole ring makes dihedral angles of 82.82 (7) and 9.92 (7)° with the 3,4-dichloro­benzene and the 3,4,5-trimethoxy­benzene ring planes, respectively. The crystal structure is stabilized by inter­molecular C—H⋯ O and C—H⋯ N hydrogen bonds. Intra­molecular C—H⋯O and C—H⋯N hydrogen bonds are also present.

## Related literature

For related literature, see: Abdel *et al.* (2003 ▶); Abdel-Rahman & Farghaly (2004 ▶); Chai *et al.* (2002 ▶); Jin *et al.* (2006 ▶); Mohd *et al.* (2004 ▶).
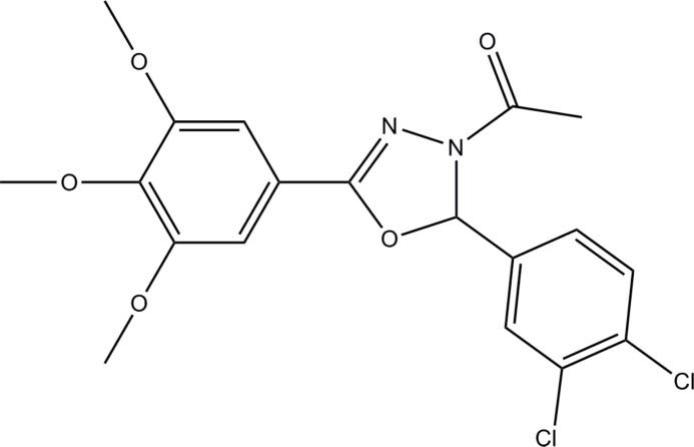


## Experimental

### 

#### Crystal data

C_19_H_18_Cl_2_N_2_O_5_
*M* *_r_* = 425.25
Monoclinic, 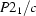

*a* = 7.6743 (4) Å
*b* = 15.9516 (8) Å
*c* = 15.7483 (8) Å
β = 90.8940 (10)°
*V* = 1927.63 (17) Å^3^
*Z* = 4
Mo *K*α radiation
μ = 0.37 mm^−1^
*T* = 173 (2) K
0.47 × 0.39 × 0.32 mm

#### Data collection

Bruker SMART 1000 CCD diffractometer
Absorption correction: multi-scan (*SADABS*; Sheldrick, 2003 ▶) *T*_min_ = 0.845, *T*_max_ = 0.890
10032 measured reflections
4159 independent reflections
3238 reflections with *I* > 2σ(*I*)
*R*_int_ = 0.025

#### Refinement

*R*[*F* ^2^ > 2σ(*F* ^2^)] = 0.041
*wR*(*F* ^2^) = 0.125
*S* = 1.04
4159 reflections
257 parameters
H-atom parameters constrained
Δρ_max_ = 0.34 e Å^−3^
Δρ_min_ = −0.31 e Å^−3^

### 

Data collection: *SMART* (Bruker, 2001 ▶); cell refinement: *SAINT-Plus* (Bruker, 2003 ▶); data reduction: *SAINT-Plus*; program(s) used to solve structure: *SHELXTL* (Sheldrick, 2008 ▶); program(s) used to refine structure: *SHELXTL*; molecular graphics: *SHELXTL*; software used to prepare material for publication: *SHELXTL*.

## Supplementary Material

Crystal structure: contains datablocks I, global. DOI: 10.1107/S1600536808020771/wn2269sup1.cif

Structure factors: contains datablocks I. DOI: 10.1107/S1600536808020771/wn2269Isup2.hkl

Additional supplementary materials:  crystallographic information; 3D view; checkCIF report

## Figures and Tables

**Table 1:** Hydrogen-bond geometry (Å, °)

*D*—H⋯*A*	*D*—H	H⋯*A*	*D*⋯*A*	*D*—H⋯*A*
C6—H6⋯O4	0.95	2.43	2.772 (2)	101
C8—H8⋯O2^i^	1.00	2.56	3.184 (3)	121
C10—H10⋯O1^ii^	0.95	2.43	3.302 (3)	153
C13—H13⋯O5^iii^	0.95	2.53	3.426 (3)	156
C16—H16*B*⋯N1	0.98	2.42	2.839 (3)	105
C18—H18*A*⋯N1^ii^	0.98	2.53	3.468 (3)	160
C18—H18*C*⋯O3	0.98	2.36	2.916 (3)	116
C19—H19*A*⋯O5^iv^	0.98	2.58	3.233 (3)	124

Symmetry codes: (i) 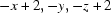; (ii) 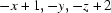; (iii) 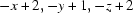; (iv) 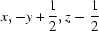.

**Fig. 1.**
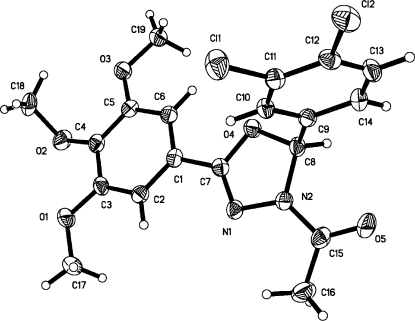
A view of the molecular structure of the title compound, showing the atom-numbering scheme. Displacement ellipsoids are drawn at the 30% probability level. Hydrogen atoms are represented by spheres of arbitrary radius.

## supplementary crystallographic information

### Comment

1,3,4-Oxadiazole derivatives are well known to possess a diverse range of bioactivities in the pharmaceutical and agrochemical fields; these include insecticidal, antibacterial, anticancer, and anti-inflammatory activities (Abdel *et al.*, 2003; Abdel-Rahman & Farghaly, 2004; Chai *et al.*, 2002; Mohd *et al.*, 2004). Here we report the synthesis and crystal structure of a 1,3,4-oxadiazole derivative containing the 3,4,5-trimethoxyphenyl unit (Fig. 1).

The bond lengths and angles in the title compound are in good agreement with expected values. Though the C8 carbon of the oxadiazole ring is *sp*^3^ hybridized, the oxadiazole ring is essentially planar. The oxadiazole ring makes dihedral angles of 82.82 (7)° and 9.92 (7)° with the 3,4-dichlorobenzene and the 3,4,5-trimethoxybenzene ring planes, respectively. These angles are somewhat different from those in a similar crystal structure (Jin *et al.*, 2006). The crystal structure exhibits intermolecular C—H··· O and C—H···N hydrogen bonds which stabilize the molecule. Intramolecular C—H···O and C—H···N hydrogen bonds are also present.

### Experimental

*N*'-(3,4-Dichlorobenzylidene)-3,4,5-trimethoxybenzohydrazide (0.38 g, 1 mmol) in acetic anhydride (8 ml) was refluxed for 2 h until the starting material disappeared, as evidenced by TLC. The resulting cool mixture was then poured into cold water, after filtration. The residue was recrystallized by slow evaporation of a methanol solution.

### Refinement

All H atoms were included in the refinement at idealized positions and refined as riding, with C—H = 0.95 (aromatic), 0.98 (methyl), 1.00Å (methine) and *U*_iso_(H) = xU_eq_(carrier atom), where *x* = 1.5 for methyl, 1.2 for all other H atoms.

### Crystal data

**Table:**

C_19_H_18_Cl_2_N_2_O_5_	*F*_000_ = 880
*M**_r_* = 425.25	*D*_x_ = 1.465 Mg m^−^^3^
Monoclinic, *P*2_1_/*c*	Mo *K*α radiation λ = 0.71073 Å
Hall symbol: -P 2ybc	Cell parameters from 5138 reflections
*a* = 7.6743 (4) Å	θ = 2.6–27.1º
*b* = 15.9516 (8) Å	µ = 0.37 mm^−^^1^
*c* = 15.7483 (8) Å	*T* = 173 (2) K
β = 90.8940 (10)º	Block, colorless
*V* = 1927.63 (17) Å^3^	0.47 × 0.39 × 0.32 mm
*Z* = 4	

### Data collection

**Table:**

Bruker SMART 1000 CCD diffractometer	4159 independent reflections
Radiation source: fine-focus sealed tube	3238 reflections with *I* > 2σ(*I*)
Monochromator: graphite	*R*_int_ = 0.025
*T* = 173(2) K	θ_max_ = 27.2º
ω scans	θ_min_ = 1.8º
Absorption correction: multi-scan(SADABS; Sheldrick, 2003)	*h* = −8→9
*T*_min_ = 0.845, *T*_max_ = 0.891	*k* = −20→18
10032 measured reflections	*l* = −13→20

### Refinement

**Table:**

Refinement on *F*^2^	Secondary atom site location: difference Fourier map
Least-squares matrix: full	Hydrogen site location: inferred from neighbouring sites
*R*[*F*^2^ > 2σ(*F*^2^)] = 0.041	H-atom parameters constrained
*wR*(*F*^2^) = 0.125	*w* = 1/[σ^2^(*F*_o_^2^) + (0.0625*P*)^2^ + 1.3473*P*] where *P* = (*F*_o_^2^ + 2*F*_c_^2^)/3
*S* = 1.04	(Δ/σ)_max_ = 0.001
4159 reflections	Δρ_max_ = 0.34 e Å^−^^3^
257 parameters	Δρ_min_ = −0.31 e Å^−^^3^
Primary atom site location: structure-invariant direct methods	Extinction correction: none

### Special details

**Table:**

Geometry. All e.s.d.'s (except the e.s.d. in the dihedral angle between two l.s. planes) are estimated using the full covariance matrix. The cell e.s.d.'s are taken into account individually in the estimation of e.s.d.'s in distances, angles and torsion angles; correlations between e.s.d.'s in cell parameters are only used when they are defined by crystal symmetry. An approximate (isotropic) treatment of cell e.s.d.'s is used for estimating e.s.d.'s involving l.s. planes.
Refinement. Refinement of *F*^2^ against ALL reflections. The weighted *R*-factor *wR* and goodness of fit *S* are based on *F*^2^, conventional *R*-factors *R* are based on *F*, with *F* set to zero for negative *F*^2^. The threshold expression of *F*^2^ > σ(*F*^2^) is used only for calculating *R*-factors(gt) *etc*. and is not relevant to the choice of reflections for refinement. *R*-factors based on *F*^2^ are statistically about twice as large as those based on *F*, and *R*- factors based on ALL data will be even larger.

### Fractional atomic coordinates and isotropic or equivalent isotropic displacement parameters (Å^2^)

**Table:**

	*x*	*y*	*z*	*U*_iso_*/*U*_eq_	
Cl1	0.29475 (7)	0.36125 (4)	0.89108 (4)	0.04132 (18)	
Cl2	0.44596 (8)	0.54237 (4)	0.85758 (4)	0.03835 (17)	
C1	0.7817 (3)	0.05267 (12)	0.99579 (13)	0.0233 (4)	
C2	0.7051 (3)	−0.00409 (13)	1.05104 (13)	0.0243 (4)	
H2	0.6667	0.0136	1.1053	0.029*	
C3	0.6857 (3)	−0.08687 (12)	1.02552 (13)	0.0254 (4)	
C4	0.7416 (3)	−0.11303 (12)	0.94532 (13)	0.0250 (4)	
C5	0.8160 (3)	−0.05492 (13)	0.89080 (12)	0.0239 (4)	
C6	0.8351 (3)	0.02847 (12)	0.91572 (13)	0.0245 (4)	
H6	0.8842	0.0683	0.8782	0.029*	
C7	0.8153 (3)	0.13859 (12)	1.02448 (13)	0.0229 (4)	
C8	0.9032 (3)	0.27301 (12)	1.00923 (13)	0.0240 (4)	
H8	1.0284	0.2903	1.0080	0.029*	
C9	0.7883 (3)	0.33955 (12)	0.97007 (12)	0.0231 (4)	
C10	0.6145 (3)	0.32243 (12)	0.95089 (13)	0.0248 (4)	
H10	0.5676	0.2685	0.9619	0.030*	
C11	0.5100 (3)	0.38450 (13)	0.91558 (13)	0.0262 (4)	
C12	0.5775 (3)	0.46408 (12)	0.90009 (13)	0.0263 (4)	
C13	0.7498 (3)	0.48130 (13)	0.91895 (14)	0.0297 (5)	
H13	0.7963	0.5354	0.9081	0.036*	
C14	0.8548 (3)	0.41901 (13)	0.95392 (13)	0.0268 (4)	
H14	0.9735	0.4308	0.9670	0.032*	
C15	0.9181 (3)	0.29242 (13)	1.16484 (14)	0.0297 (5)	
C16	0.8863 (4)	0.25497 (16)	1.25067 (15)	0.0428 (6)	
H16A	0.9930	0.2277	1.2717	0.064*	
H16B	0.7925	0.2134	1.2461	0.064*	
H16C	0.8525	0.2993	1.2902	0.064*	
C17	0.5778 (3)	−0.12792 (16)	1.16051 (15)	0.0383 (6)	
H17A	0.6858	−0.1100	1.1892	0.058*	
H17B	0.5312	−0.1774	1.1893	0.058*	
H17C	0.4923	−0.0824	1.1623	0.058*	
C18	0.6289 (3)	−0.22110 (15)	0.85465 (16)	0.0368 (5)	
H18A	0.5145	−0.1941	0.8580	0.055*	
H18B	0.6143	−0.2821	0.8544	0.055*	
H18C	0.6861	−0.2036	0.8024	0.055*	
C19	0.9654 (3)	−0.03053 (15)	0.76156 (14)	0.0351 (5)	
H19A	0.8912	0.0169	0.7450	0.053*	
H19B	1.0019	−0.0607	0.7106	0.053*	
H19C	1.0685	−0.0098	0.7925	0.053*	
N1	0.7969 (2)	0.16636 (10)	1.09988 (11)	0.0248 (4)	
N2	0.8524 (2)	0.25043 (10)	1.09633 (10)	0.0251 (4)	
O1	0.6131 (2)	−0.14829 (9)	1.07411 (10)	0.0345 (4)	
O2	0.7344 (2)	−0.19688 (9)	0.92680 (10)	0.0354 (4)	
O3	0.8701 (2)	−0.08591 (9)	0.81492 (9)	0.0304 (3)	
O4	0.87792 (19)	0.19385 (8)	0.96599 (9)	0.0265 (3)	
O5	0.9955 (2)	0.35859 (10)	1.15404 (11)	0.0401 (4)	

### Atomic displacement parameters (Å^2^)

**Table:**

	*U*^11^	*U*^22^	*U*^33^	*U*^12^	*U*^13^	*U*^23^
Cl1	0.0272 (3)	0.0402 (3)	0.0563 (4)	−0.0036 (2)	−0.0087 (2)	0.0089 (3)
Cl2	0.0433 (3)	0.0298 (3)	0.0419 (3)	0.0081 (2)	−0.0035 (2)	0.0093 (2)
C1	0.0220 (9)	0.0187 (9)	0.0290 (10)	0.0012 (8)	−0.0052 (8)	0.0011 (8)
C2	0.0229 (10)	0.0251 (10)	0.0249 (10)	0.0007 (8)	−0.0009 (8)	−0.0016 (8)
C3	0.0252 (10)	0.0206 (10)	0.0303 (11)	−0.0032 (8)	−0.0026 (8)	0.0056 (8)
C4	0.0283 (10)	0.0160 (9)	0.0307 (11)	0.0004 (8)	−0.0043 (8)	0.0000 (8)
C5	0.0243 (10)	0.0225 (10)	0.0247 (10)	0.0030 (8)	−0.0026 (8)	−0.0024 (7)
C6	0.0255 (10)	0.0217 (10)	0.0261 (10)	0.0000 (8)	−0.0034 (8)	0.0027 (8)
C7	0.0217 (9)	0.0210 (10)	0.0260 (10)	0.0009 (8)	−0.0011 (7)	0.0024 (7)
C8	0.0276 (10)	0.0172 (9)	0.0273 (10)	−0.0030 (8)	0.0015 (8)	−0.0023 (7)
C9	0.0280 (10)	0.0194 (9)	0.0219 (9)	0.0003 (8)	0.0017 (8)	−0.0018 (7)
C10	0.0277 (10)	0.0201 (10)	0.0266 (10)	−0.0032 (8)	0.0012 (8)	0.0004 (8)
C11	0.0243 (10)	0.0283 (11)	0.0261 (10)	−0.0029 (8)	0.0004 (8)	0.0007 (8)
C12	0.0328 (11)	0.0211 (10)	0.0250 (10)	0.0042 (8)	0.0020 (8)	0.0035 (8)
C13	0.0363 (12)	0.0201 (10)	0.0328 (11)	−0.0040 (9)	0.0030 (9)	0.0025 (8)
C14	0.0257 (10)	0.0216 (10)	0.0332 (11)	−0.0039 (8)	0.0016 (8)	−0.0016 (8)
C15	0.0363 (12)	0.0215 (10)	0.0312 (11)	0.0032 (9)	−0.0006 (9)	−0.0070 (8)
C16	0.0668 (17)	0.0339 (13)	0.0277 (12)	−0.0001 (12)	−0.0001 (11)	−0.0065 (10)
C17	0.0478 (14)	0.0373 (13)	0.0300 (12)	−0.0120 (11)	0.0034 (10)	0.0064 (10)
C18	0.0350 (12)	0.0305 (12)	0.0448 (13)	−0.0101 (10)	−0.0005 (10)	−0.0107 (10)
C19	0.0431 (13)	0.0345 (12)	0.0279 (11)	−0.0098 (10)	0.0052 (9)	−0.0017 (9)
N1	0.0291 (9)	0.0177 (8)	0.0277 (9)	−0.0016 (7)	−0.0018 (7)	−0.0001 (7)
N2	0.0309 (9)	0.0199 (8)	0.0245 (9)	−0.0015 (7)	0.0001 (7)	−0.0013 (7)
O1	0.0480 (10)	0.0232 (8)	0.0325 (8)	−0.0107 (7)	0.0041 (7)	0.0023 (6)
O2	0.0520 (10)	0.0185 (7)	0.0355 (9)	−0.0018 (7)	−0.0072 (7)	−0.0029 (6)
O3	0.0388 (9)	0.0246 (8)	0.0278 (8)	−0.0043 (6)	0.0048 (6)	−0.0035 (6)
O4	0.0367 (8)	0.0168 (7)	0.0262 (7)	−0.0013 (6)	0.0029 (6)	−0.0010 (5)
O5	0.0531 (10)	0.0255 (8)	0.0416 (10)	−0.0062 (7)	−0.0027 (8)	−0.0096 (7)

### Geometric parameters (Å, °)

**Table:**

Cl1—C11	1.730 (2)	C11—C12	1.394 (3)
Cl2—C12	1.734 (2)	C12—C13	1.378 (3)
C1—C6	1.387 (3)	C13—C14	1.388 (3)
C1—C2	1.392 (3)	C13—H13	0.9500
C1—C7	1.465 (3)	C14—H14	0.9500
C2—C3	1.388 (3)	C15—O5	1.224 (3)
C2—H2	0.9500	C15—N2	1.360 (3)
C3—O1	1.367 (2)	C15—C16	1.501 (3)
C3—C4	1.404 (3)	C16—H16A	0.9800
C4—O2	1.370 (2)	C16—H16B	0.9800
C4—C5	1.392 (3)	C16—H16C	0.9800
C5—O3	1.364 (2)	C17—O1	1.429 (3)
C5—C6	1.394 (3)	C17—H17A	0.9800
C6—H6	0.9500	C17—H17B	0.9800
C7—N1	1.277 (3)	C17—H17C	0.9800
C7—O4	1.368 (2)	C18—O2	1.438 (3)
C8—O4	1.446 (2)	C18—H18A	0.9800
C8—N2	1.476 (3)	C18—H18B	0.9800
C8—C9	1.506 (3)	C18—H18C	0.9800
C8—H8	1.0000	C19—O3	1.429 (3)
C9—C10	1.390 (3)	C19—H19A	0.9800
C9—C14	1.391 (3)	C19—H19B	0.9800
C10—C11	1.385 (3)	C19—H19C	0.9800
C10—H10	0.9500	N1—N2	1.409 (2)
			
C6—C1—C2	121.40 (18)	C12—C13—H13	120.3
C6—C1—C7	119.20 (18)	C14—C13—H13	120.3
C2—C1—C7	119.32 (18)	C13—C14—C9	120.8 (2)
C3—C2—C1	118.83 (19)	C13—C14—H14	119.6
C3—C2—H2	120.6	C9—C14—H14	119.6
C1—C2—H2	120.6	O5—C15—N2	119.3 (2)
O1—C3—C2	124.24 (19)	O5—C15—C16	123.7 (2)
O1—C3—C4	115.06 (18)	N2—C15—C16	117.0 (2)
C2—C3—C4	120.70 (18)	C15—C16—H16A	109.5
O2—C4—C5	122.30 (19)	C15—C16—H16B	109.5
O2—C4—C3	118.03 (18)	H16A—C16—H16B	109.5
C5—C4—C3	119.42 (18)	C15—C16—H16C	109.5
O3—C5—C4	115.61 (18)	H16A—C16—H16C	109.5
O3—C5—C6	124.10 (19)	H16B—C16—H16C	109.5
C4—C5—C6	120.27 (19)	O1—C17—H17A	109.5
C1—C6—C5	119.37 (19)	O1—C17—H17B	109.5
C1—C6—H6	120.3	H17A—C17—H17B	109.5
C5—C6—H6	120.3	O1—C17—H17C	109.5
N1—C7—O4	116.60 (17)	H17A—C17—H17C	109.5
N1—C7—C1	126.14 (18)	H17B—C17—H17C	109.5
O4—C7—C1	117.22 (17)	O2—C18—H18A	109.5
O4—C8—N2	100.90 (14)	O2—C18—H18B	109.5
O4—C8—C9	110.42 (16)	H18A—C18—H18B	109.5
N2—C8—C9	113.01 (16)	O2—C18—H18C	109.5
O4—C8—H8	110.7	H18A—C18—H18C	109.5
N2—C8—H8	110.7	H18B—C18—H18C	109.5
C9—C8—H8	110.7	O3—C19—H19A	109.5
C10—C9—C14	119.51 (18)	O3—C19—H19B	109.5
C10—C9—C8	120.30 (18)	H19A—C19—H19B	109.5
C14—C9—C8	120.18 (18)	O3—C19—H19C	109.5
C11—C10—C9	119.63 (19)	H19A—C19—H19C	109.5
C11—C10—H10	120.2	H19B—C19—H19C	109.5
C9—C10—H10	120.2	C7—N1—N2	104.79 (16)
C10—C11—C12	120.43 (19)	C15—N2—N1	123.09 (17)
C10—C11—Cl1	118.75 (16)	C15—N2—C8	121.12 (17)
C12—C11—Cl1	120.82 (16)	N1—N2—C8	110.70 (15)
C13—C12—C11	120.13 (19)	C3—O1—C17	117.06 (17)
C13—C12—Cl2	119.37 (16)	C4—O2—C18	116.81 (17)
C11—C12—Cl2	120.49 (17)	C5—O3—C19	117.18 (16)
C12—C13—C14	119.45 (19)	C7—O4—C8	106.95 (15)
			
C6—C1—C2—C3	1.4 (3)	C10—C11—C12—Cl2	−178.89 (16)
C7—C1—C2—C3	−175.26 (18)	Cl1—C11—C12—Cl2	1.2 (3)
C1—C2—C3—O1	179.76 (19)	C11—C12—C13—C14	−0.4 (3)
C1—C2—C3—C4	−0.2 (3)	Cl2—C12—C13—C14	179.24 (16)
O1—C3—C4—O2	−6.1 (3)	C12—C13—C14—C9	0.0 (3)
C2—C3—C4—O2	173.88 (18)	C10—C9—C14—C13	0.0 (3)
O1—C3—C4—C5	179.51 (18)	C8—C9—C14—C13	−179.09 (19)
C2—C3—C4—C5	−0.5 (3)	O4—C7—N1—N2	−0.3 (2)
O2—C4—C5—O3	4.6 (3)	C1—C7—N1—N2	177.36 (18)
C3—C4—C5—O3	178.68 (18)	O5—C15—N2—N1	165.36 (19)
O2—C4—C5—C6	−174.01 (18)	C16—C15—N2—N1	−16.2 (3)
C3—C4—C5—C6	0.1 (3)	O5—C15—N2—C8	12.9 (3)
C2—C1—C6—C5	−1.8 (3)	C16—C15—N2—C8	−168.66 (19)
C7—C1—C6—C5	174.89 (18)	C7—N1—N2—C15	−153.3 (2)
O3—C5—C6—C1	−177.46 (18)	C7—N1—N2—C8	1.6 (2)
C4—C5—C6—C1	1.0 (3)	O4—C8—N2—C15	153.32 (18)
C6—C1—C7—N1	−169.31 (19)	C9—C8—N2—C15	−88.8 (2)
C2—C1—C7—N1	7.4 (3)	O4—C8—N2—N1	−2.2 (2)
C6—C1—C7—O4	8.3 (3)	C9—C8—N2—N1	115.70 (18)
C2—C1—C7—O4	−174.95 (17)	C2—C3—O1—C17	−9.0 (3)
O4—C8—C9—C10	45.1 (2)	C4—C3—O1—C17	171.03 (19)
N2—C8—C9—C10	−67.1 (2)	C5—C4—O2—C18	−65.1 (3)
O4—C8—C9—C14	−135.80 (19)	C3—C4—O2—C18	120.8 (2)
N2—C8—C9—C14	112.0 (2)	C4—C5—O3—C19	−172.83 (18)
C14—C9—C10—C11	0.3 (3)	C6—C5—O3—C19	5.7 (3)
C8—C9—C10—C11	179.43 (18)	N1—C7—O4—C8	−1.2 (2)
C9—C10—C11—C12	−0.7 (3)	C1—C7—O4—C8	−179.02 (17)
C9—C10—C11—Cl1	179.22 (15)	N2—C8—O4—C7	1.92 (19)
C10—C11—C12—C13	0.7 (3)	C9—C8—O4—C7	−117.85 (17)
Cl1—C11—C12—C13	−179.17 (16)		

### Hydrogen-bond geometry (Å, °)

**Table:**

*D*—H···*A*	*D*—H	H···*A*	*D*···*A*	*D*—H···*A*
C6—H6···O4	0.95	2.43	2.772 (2)	101
C8—H8···O2^i^	1.00	2.56	3.184 (3)	121
C10—H10···O1^ii^	0.95	2.43	3.302 (3)	153
C13—H13···O5^iii^	0.95	2.53	3.426 (3)	156
C16—H16B···N1	0.98	2.42	2.839 (3)	105
C18—H18A···N1^ii^	0.98	2.53	3.468 (3)	160
C18—H18C···O3	0.98	2.36	2.916 (3)	116
C19—H19A···O5^iv^	0.98	2.58	3.233 (3)	124

Symmetry codes: (i) −*x*+2, −*y*, −*z*+2; (ii) −*x*+1, −*y*, −*z*+2; (iii) −*x*+2, −*y*+1, −*z*+2; (iv) *x*, −*y*+1/2, *z*−1/2.
